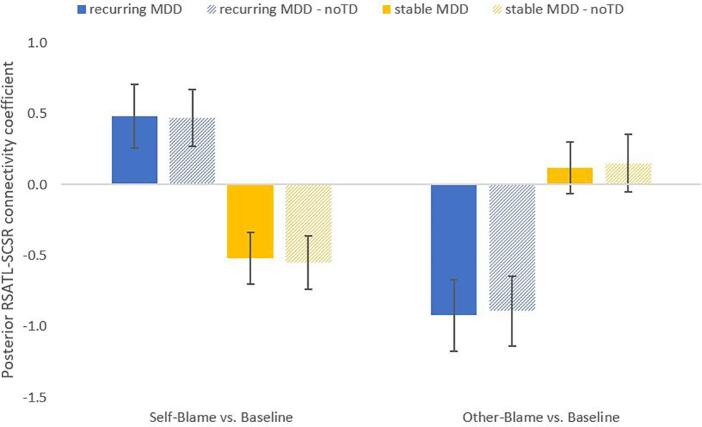# Internal reliability of blame-related functional MRI measures in major depressive disorder

**DOI:** 10.1016/j.nicl.2021.102901

**Published:** 2021-11-28

**Authors:** Diede Fennema, Owen O'Daly, Gareth J. Barker, Jorge Moll, Roland Zahn

**Affiliations:** a Department of Psychological Medicine, Institute of Psychiatry, Psychology & Neuroscience, Centre for Affective Disorders, King’s College London, London SE5 8AZ, UK; b Department of Neuroimaging, Institute of Psychiatry, Psychology & Neuroscience, King’s College London, London, UK; c Cognitive and Behavioural Neuroscience Unit, D’Or Institute for Research and Education (IDOR), 22280-080 Rio de Janeiro, RJ, Brazil; d National Service for Affective Disorders, South London and Maudsley NHS Foundation Trust, London SE5 8AZ, UK

**Keywords:** fMRI, Split-half reliability, Internal consistency, Intraclass coefficient correlation, Self-blame, Depression

## Abstract

**Background:**

In major depressive disorder (MDD), self-blame-related fMRI measures have shown the potential to be used as prognostic markers for recurrence risk. Like most potential fMRI markers, however, their reliability is unclear. Here, we probed the internal reliability of self-blame-related fMRI measures, as well as the impact of different modelling approaches on reliability metrics and validity.

**Methods:**

Internal consistency (i.e. split-half reliability) was calculated for blood oxygen level-dependent (BOLD) responses and psychophysiological interactions (PPI) related to self-blame-related biases in medication-free remitted MDD participants (n = 81) and healthy controls (n = 41). Trial-length was modelled using three durations (0, 2 and 5 s), which was convolved with the haemodynamic response function (HRF) with and without time and dispersion derivatives. Intraclass correlation coefficients (ICCs) were calculated for simple contrasts examining activation to self-blaming emotions and other-blaming emotions and the more complex contrast of the subtraction-based difference between self- and other-blaming emotions within the following *a priori* ROIs: right superior anterior temporal lobe seed region, anterior subgenual cingulate cortex, posterior subgenual cortex and right striatum / pallidum.

**Results:**

Across ROIs, we obtained fair reliability (ICC ≥ 0.40) for simple, but poor reliability (ICC < 0.40) for more complex fMRI measures related to self-blame. Despite this low internal consistency of complex measures at the individual level, we observed robust activation at the group-level, reproducing previously published results.

**Conclusions:**

While simple BOLD contrasts had fair reliability, previously employed PPI models had poor reliability and simple connectivity measures lacked predictive validity. This calls for the development of functional connectivity measures that strike a better balance between reliability and validity for future clinical applications, which require robust measures at the individual rather than group-level.

## Highlights

• Self-blame-related fMRI measures were previously validated in depressive disorders.
• Reproducibility and internal consistency as a measure of reliability were examined.
• Whilst simple fMRI measures exhibited fair reliability, complex measures did not.
• Yet, complex measures showed reproducible clinical validity at the group level.
• Connectivity measures, that balance reliability and validity better, are needed.

## 1 Introduction

In the field of neuroimaging, particularly functional magnetic resonance imaging (fMRI), reliability and validity of measures have come under increased scrutiny (Bennett and Miller, 2010, Cremers et al., 2017, Noble et al., 2019, Specht, 2020). A recent meta-analysis of common task-based fMRI measures suggests overall poor reliability regardless of task design, task length or region-of-interest (ROI) (Elliott et al., 2020). This poses a challenge in the quest for imaging biomarkers in psychiatry, especially if these are intended for prognostic purposes or predicting response to treatment (Bennett and Miller, 2010, Specht, 2020). In major depressive disorder (MDD), self-blame-related fMRI measures have shown the potential to be used as prognostic markers for recurrence risk (Lawrence et al., 2021). Like for most potential fMRI markers, however, their reliability is unclear and this may affect their clinical usefulness (Bennett and Miller, 2010, Fournier et al., 2014, Dubois and Adolphs, 2016, Nord et al., 2017). Low reliability can also introduce biases when investigating associations between fMRI measures and other clinical measures, for example inflating effect sizes or falsely rejecting a hypothesis (Elliott et al., 2020, Fournier et al., 2014).

One commonly used measure for estimating reliability is the intraclass correlation coefficient (ICC), which gives an index of similarity across repeated measurements (Shrout and Fleiss, 1979). There are ten variations of ICC, with each variant differing in their assumptions, calculations and interpretations (McGraw and Wong, 1996, Koo and Li, 2016), but they all represent a ratio of between-subject to within-subject variability and reflect what proportion of variance can be attributed to differences among subjects (Hedge et al., 2018). However, in neuroimaging, some of the variance of the blood oxygen level-dependent (BOLD) signal, an indirect measure of neural activity, cannot be reproduced across sessions and introduces “noise” related to, for example, variations in signal-to-noise ratio of the scanner, subject motion, subject physiology or cognitive state (Bennett and Miller, 2010, Dubois and Adolphs, 2016, Plichta et al., 2012). These sources of noise are major contributors to both within- and between-subject variance and consequently if, following pre-processing and fixed-effects modelling, a large unstable residual variance remains, it is unlikely that it will give consistent results under similar experimental conditions and, thus, will be limited in its validity (Fournier et al., 2014, Dubois and Adolphs, 2016).

Reliability metrics like ICC are dependent on the modelling approach, such as choice of haemodynamic response function (HRF), choice of regressors and definition of contrasts (Fournier et al., 2014, Caceres et al., 2009, Gorgolewski et al., 2013, Di and Biswal, 2017). For instance, event-related designs assume that neural activity occurs for short and discrete intervals, which can be uniformly modelled as a HRF with a fixed shape (Handwerker et al., 2004, Grinband et al., 2008). Even though the use of the canonical HRF typically facilitates the detection of task-related fMRI activity, it fails to consider subtle BOLD signal variability across brain regions and subjects that can convey important information about the intensity, timing and duration of underlying brain activity (Handwerker et al., 2004, Grinband et al., 2008, Lindquist and Wager, 2007, Handwerker et al., 2012, Bonakdarpour et al., 2007). Modelling approaches for the HRF differ in their specificity and sensitivity of capturing these parameters (Lindquist and Wager, 2007, Lindquist et al., 2009). As a result, the HRF might not adequately capture temporal and spatial variability, which in turn could have an impact on the reliability observed (Handwerker et al., 2004, Lindquist and Wager, 2007, Lindquist et al., 2009, Calhoun et al., 2004).

In addition to regional BOLD activation, functional connectivity (i.e. correlations between neural activity of brain areas) (Friston, 2011, O’Reilly et al., 2012) are of high interest as potential biomarkers in psychiatric disorders. Psychophysiological interaction (PPI) analysis examines the interaction between influences from task-related factors (i.e. psychological) and observed brain activity (i.e. physiological), allowing for inferences about functional integration or interactions between cortical areas (Friston et al., 1997). A more recent variant of PPI is known as generalised PPI (gPPI) (McLaren et al., 2012). Like standard PPI, it computes the interaction between a seed BOLD time-series and a condition-specific interaction factor, but it includes the interaction factors from all conditions simultaneously, as opposed to only modelling two regressors (McLaren et al., 2012). Even when a condition might be irrelevant, modelling the entire experimental space offers greater sensitivity and specificity than restricting the PPI to a single pair of regressors (McLaren et al., 2012, Cisler et al., 2014). However, few studies have investigated whether this is reflected in reliability estimates of gPPI when compared to standard PPI.

More and more studies are reporting on test–retest reliability, albeit with a limited focus on clinical populations and relatively small samples (Bennett and Miller, 2010, Elliott et al., 2020). Test-retest reliability places an emphasis on the relative stability of fMRI measures over time and is sensitive to between-session variations, such as scanner noise and participant state (Bennett and Miller, 2010). If the neural measure is relatively stable across repeated sessions, it might be reflecting trait-like cognitive processes (Hajcak et al., 2017). However, this type of reliability does not address the internal consistency of a measure, i.e. how consistent it is within a session (Luking, 2017). In fact, high test–retest reliability does not guarantee good internal consistency as it could reflect multiple unrelated processes rather than a specific trait (Luking, 2017). Conversely, if a measure captures a state variable, it could have good internal consistency but poor test–retest reliability (Luking, 2017).

Even though internal consistency places a limit on validity of a measure and on its sensitivity to detect individual difference effects (Specht, 2020, Hajcak et al., 2017, Feldt, 1997), few studies report on the internal consistency of fMRI measures. To the authors’ knowledge, four studies have specifically reported on the internal consistency of task-related fMRI measures using a split-half method: BOLD response to an emotional face-matching task (Infantolino et al., 2018, Gianaros et al., 2020), BOLD response to a monetary gain and loss feedback task (Luking, 2017), and brain activation maps in response to an intertemporal choice task (Fröhner et al., 2019). Most test–retest reliability studies report on within-session reliability, which can be interpreted as a measure of internal consistency if there is a very short interval between runs (Korucuoglu, 2021). However, reliability estimates tend to decline with increasing test–retest interval duration (Korucuoglu, 2021, Bennett and Miller, 2013), and the emphasis is on the temporal stability of the overall neural measure rather than the homogeneity of trials making up the neural measure (Hajcak et al., 2017).

Here, we investigated the internal consistency of an fMRI paradigm designed to measure self-blame-related biases in MDD, for which we have shown internal validity as predictive of recurrence risk (Lythe et al., 2015, Lawrence et al., 2021). This paradigm is based on the revised learned helplessness model, which proposes that vulnerability to MDD could be explained by a tendency to attribute failure to stable, global and internal factors, i.e. for patients to blame themselves, resulting in overgeneralised self-blame (Abramson et al., 1978). It has highlighted the role of the subgenual region, which showed increased BOLD signal in guilt-prone people and those with a history of MDD (Lythe et al., 2020) and its self-blame-selective hyper-connectivity with the right superior anterior temporal lobe (RSATL) was associated with recurrence risk (Lythe et al., 2015). Moreover, the paradigm identified a strong recurrence predicting effect of right striatum/pallidum hyper-connectivity with the RSATL (Lythe et al., 2015, Lawrence et al., 2021).

**Supplementary figure 1:**
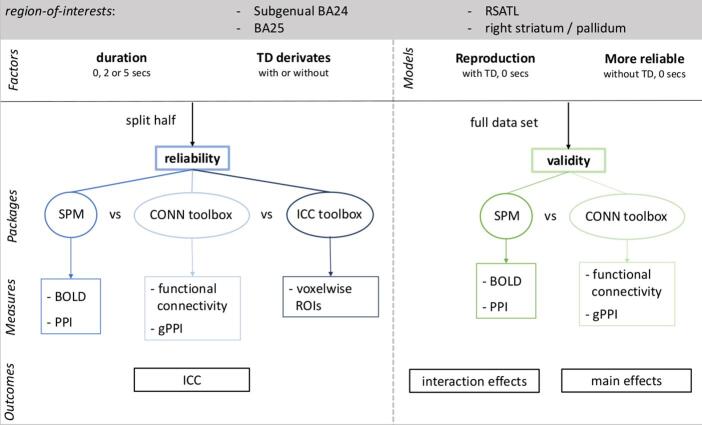


However, it is unknown to what extent these measures are reliable. Thus, the aim of this study was to probe the internal reliability of self-blame-related fMRI measures, using regions-of-interest based on these previous findings: RSATL seed region, anterior subgenual cingulate cortex, posterior subgenual cortex and right striatum / pallidum (Green et al., 2012, Lythe et al., 2015, Lawrence et al., 2021, Lythe et al., 2020). Given the potential influence of modelling approaches on reliability metrics, we examined the impact of different durations, modelling with and without time and dispersion derivatives, and comparing standard PPI with gPPI. Moreover, we sought to test whether more reliable fMRI measures exhibit clinical validity by examining their association with MDD (comparing remitted MDD vs. control participants with no personal or family history of MDD) and recurrence risk. Recurrence risk was captured longitudinally by comparing medication-free remitted MDD at baseline who remained stable (stable MDD) over the subsequent 14 months of clinical follow-up with those who developed a recurring episode (recurring MDD). Please see Supplementary Figure 1 for an overview of the approach taken to investigate reliability and validity of self-blame-related fMRI measures.

## 2 Methods and materials

### 2.1 Participants

The fMRI dataset used to assess reliability and reproducibility was collected as part of a previously published longitudinal study, which examined whether self-blame-selective alterations in anterior temporal fMRI connectivity predict subsequent recurrence of depression (see Lythe et al., 2015). Ethical approval was obtained from the South Manchester National Health Service Research Ethics Committee. All participants provided informed consent and received compensation for their time and travel expenses.

Participants in the remitted MDD group fulfilled criteria for MDD according to Diagnostic and Statistical Manual of Mental Disorders (DSM-IV-TR; APA, 2000) and were in remission for at least six months. The main exclusion criteria were current Axis-I disorders, including a history of substance or alcohol abuse and past comorbid Axis-I disorders being the likely cause of depressive symptoms. Participants were followed up clinically at 3, 6 and 14 months either in person or over the phone using the Longitudinal Interval Follow-up Evaluation (Keller et al., 1987). The control group (HC) had no current or past Axis-I diagnoses and no first-degree family history of MDD, bipolar disorder or schizophrenia. Both groups were psychotropic medication free, right-handed, native English speaking, with normal or corrected-to-normal vision.

For the primary imaging analysis, 109 participants were included (remitted MDD = 70 and HC = 39, respectively), meeting strict criteria for signal dropout (sufficient coverage of the subgenual cingulate cortex and adjacent septal region) and movement (translation < 7 mm or rotation < 5 degrees). An additional thirteen participants, who did not meet the strictest quality control threshold, were included in further analysis (remitted MDD = 11 and HC = 2, respectively). Please see Table 1 for demographic, basic clinical and fMRI characteristics for the participants included.

**Table 1:** Demographic, basic clinical and fMRI characteristics.

	remitted MDD	HC	Total	Comparison MDD vs HC
	n = 81	n = 41	n = 122	
Sex	25 male / 56 female	15 male / 26 female	40 male / 82 female	χ^2^ (1,122) = 0.404, *p* = .53
Age in years	34.4 ± 12.5; 18–63	33.3 ± 13.1; 20–64	34.0 ± 12.6; 18–64	*t*(120) = 0.46, *p* = .64
Years of education	16.7 ± 2.4; 12–22	17.3 ± 2.5; 12–25	16.9 ± 2.4; 12–25	*t*(120) = −1.29, *p* = .20
BDI score	4.0 ± 4.1; 0–17	0.9 ± 1.7; 0–6	3.0 ± 3.8; 0–17	*t*(117.5) = 5.84, *p* < .001
MADRS	1.2 ± 1.6; 0–6	0.6 ± 1.2; 0–4	1.0 ± 1.5; 0–6	*t*(101.7) = 2.33, *p =* .02
GAF	85.2 ± 5.7; 70–90	89.0 ± 2.7; 80–91	86.5 ± 5.2; 70–91	*t*(119.6) = −4.97, *p* < .001
RMS translation	0.34 ± 0.17	0.36 ± 0.18		*t*(120) = −0.76, *p =* .45
RMS rotation	0.01 ± 0.00	0.01 ± 0.00		*t*(120) = 0.75, *p =* .45
MDD = major depressive disorder; HC = healthy control; BDI = Beck Depression Inventory; MADRS = Montgomery-Åsberg Depression Rating Scale; GAF = Global Assessment of Functioning Scale; RMS = root mean square. Means, standard deviations and range are reported (*M ± SD; minimum – maximum).*				

### 2.2 fMRI data acquisition

As previously described by Green and colleagues (Green et al., 2012), an fMRI protocol optimised for detection of ventral brain regions was used. T2*-weighted echo-planar images (3 runs of 405 volumes with 5 dummy scans, repetition time = 2000 ms) and T1-weighted, magnetisation-prepared, rapid-acquisition gradient-echo structural images were acquired on an MRI scanner (3T Achieva, Philips, see Supplementary Methods for more details on data acquisition).

**Supplementary figure 2:**
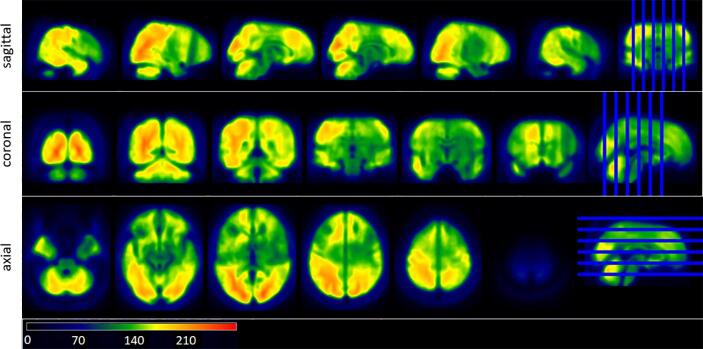


As demonstrated by measurements of the temporal signal-to-noise, i.e. “the mean of a voxel’s BOLD signal over time divided by its standard deviation over time” (Welvaert et al., 2013), overall signal quality was very good (see Supplementary Figure 2).

### 2.3 fMRI paradigm

The fMRI task has been described in detail previously (Lythe et al., 2015, Lythe et al., 2020, Green et al., 2012). In short, participants were shown written statements describing hypothetical social behaviours, in which either the participant (self-agency) or their best friend (other-agency) acts counter to social and moral values (e.g. impatient, dishonest). In the self-agency condition, the participant acts towards their best friend (number of trials = 90), while in the other-agency their best friend acts towards them (number of trials = 90). Self- and other-agency conditions contained the same social concepts, for instance, “[participant’s name] does act dishonestly towards [best friend’s name]” (self-agency) and “[best friend’s name] does act dishonestly towards [participant’s name]” (other-agency).

Stimuli were presented for five seconds, followed by a jittered inter-trial interval with a mean duration of four seconds, and participants were asked to report how unpleasant they would find this (i.e. the described behaviour; “mildly” or “very”) via a button press. A baseline visual fixation pattern (number of trials = 90) was pseudo-randomly interspersed across three runs, the order of which was counterbalanced across participants. For more details, please see (Lythe et al., 2015).

After the scanning session, participants rated the degree of unpleasantness associated with each stimulus on a 7-point Likert scale (1 = not unpleasant, 7 = extremely unpleasant). Self- and other-blaming emotion trials were defined as those that were perceived as highly unpleasant (those rated post-scanning at individual median or above) in the respective self- and other-agency conditions.

### 2.4 fMRI analysis

Pre-processed functional images were made available by the original researchers; these had been realigned, warped, co-registered to the participant’s T1-weighted images, normalised, and smoothed with a kernel of 6mm full-width-half-maximum. Each participant’s batch file was manually checked for the order of runs and the vector onsets against raw E-Prime files (Psychology Software Tools, Pittsburgh, PA). Seven participants showed minor discrepancies in the order of runs and vector onsets, which were corrected before the modelling.

#### 2.4.1 fMRI analysis: split-half reliability

To keep the analysis consistent with previously published reports of the dataset (Lythe et al., 2015, Lythe et al., 2020), Statistical Parametric Mapping (SPM8; http://www.fil.ion.ucl.ac.uk/spm8) was used to determine the reliability of the BOLD- and PPI-signals. The CONN toolbox v17.c (www.nitrc.org/projects/conn, RRID:SCR_009550), an SPM-based toolbox, was used to determine the reliability of functional and effective connectivity measures.

The set-up of the fMRI paradigm, which presents a relatively large number of self- and other-blaming emotion trials (number of trials = 90 each) in the same testing session, lends itself to assessment of internal consistency using the split-half method. This involves splitting the trials for each subject in two halves and subsequently comparing the similarity of the neural measure between the two halves (Luking, 2017). As the neural measures are derived from the same task at the same testing session, the two halves should be strongly correlated if the task is internally consistent, i.e. measure the same construct at a similar level of precision. Using the split-half method, the data were split into two halves based on even and odd trials, with no significant difference in distribution of the highly unpleasant (rated individual median or above) and low unpleasantness (rated below individual median) stimuli caused by the variation of individual unpleasantness ratings (χ^2^_high_ (1, number of ratings = 12817) = 0.01, *p* > .05; χ^2^_low_ (1, number of ratings = 9083) = 0.00, *p* > .05).

For the BOLD analysis, even and odd trials of each condition (highly unpleasant self-agency, low unpleasantness self-agency, highly unpleasant other-agency, low unpleasantness other-agency and null event (i.e. visual fixation trial)) were modelled at the first level, with 15 trials of each condition included in each split half. Movement parameters (i.e. six parameters describing movement by rotation and translation in three dimensions each) were included as covariates, and temporal and spatial derivatives of the haemodynamic response function were modelled with an event duration of 0 s. This model follows the original model parameters as reported in (Lythe et al., 2020).

In addition, alternative BOLD models were run to explore the influence of modelling without time and dispersion derivatives and with trials modelled with a varying duration on reliability. Event-related designs assume that neural activity occurs for short and discrete intervals and aim to measure transient changes in brain activity (Grinband et al., 2008). However, the haemodynamic response is variable across different brain regions (Handwerker et al., 2004). The duration modelled reflects the assumed duration of neural activity in the brain regions of interest.

Here, we opted to model trial-length using three different durations (0, 2 and 5 s). Generally, events in an event-related design are assumed to have zero duration (duration = 0 s; (Grinband et al., 2008). However, with a mean duration of 5 s per stimulus, it might be more appropriate to model the full trial period to capture all processes (duration = 5 s), particularly as the precise timing of individual processes, e.g. emotion and visual processing, is unclear and likely significantly overlap. Unpublished electroencephalography (EEG) data on the value-related moral sentiment task suggests emotional aspects of the stimuli being detected between 0 and 2 s (duration = 2 s). See Supplementary Methods for more details on duration. A final BOLD model, based on the most reliable model, examined the impact of a temporal split of the data, i.e. first and second half of trials, as opposed to odd and even trials used as our primary method.

For the PPI analysis, the signal from the seed region as used in (Lythe et al., 2015), i.e. the right superior anterior temporal lobe (RSATL; MNI coordinates x = 58, y = 0, z = −12; 6 mm sphere), was extracted. Interaction terms were created for odd and even trials, which is the multiplication of the psychological variable (the main effects of the conditions, i.e. self-agency vs fixation and other-agency vs fixation) with the physiological variable (the RSATL signal time course irrespective of condition).

Using the BOLD- and PPI-models, contrasts were created to examine activation to self-blaming emotions (self-agency vs fixation), other-blaming emotions (other-agency vs fixation) and the subtraction-based difference between self- and other-blaming emotions for each split half. The MarsBaR toolbox (Brett et al., 2002) was used to extract the mean BOLD- and PPI-response for each contrast, i.e. difference between beta values, for individual participants within the following regions-of-interest derived from our previous work as being most relevant for self-blaming biases and recurrence risk in MDD:

1. Right superior anterior temporal lobe seed region, as used in Green et al. (2012) and Lythe et al. (2015). MNI coordinates: x = 58, y = 0, z = −12; 6 mm sphere
2. Anterior subgenual cingulate cortex (BA24), as used as an ROI in Green et al. (2012). MNI coordinates: x = −4, y = 23, z = −5; 6 mm sphere
3. Posterior subgenual cortex (BA25), resulting from Lythe et al. (2015). MNI coordinates: x  = 1, y = 15, z = −7; 6 mm sphere
4. Right striatum / pallidum, right hemispheric part of our a priori basal ganglia ROI used in Green et al. (2012) and Lawrence et al., 2021). MNI coordinates: x = 21, y = 6, z = 4; 6 mm sphere

The CONN toolbox v17.c was used to explore alternative normal seed-based connectivity and PPI measures. Pre-processed data were imported into the toolbox, and stimuli onsets and duration for the conditions (i.e. self-agency, other-agency and null events) were specified. Raw BOLD signal was used as analysis unit instead of the default percent signal change to reflect the use of pre-processed data. Motion parameters and confounding temporal covariates were removed via CONNs CompCor algorithm (Whitfield-Gabrieli and Nieto-Castanon, 2012).

Reliability was assessed for both standard functional connectivity (weighted generalised linear model (GLM)) and gPPI models. Simple ROI-to-ROI analyses were performed to determine the functional connectivity between a seed region, i.e. the RSATL (MNI coordinates x = 58, y = 0, z = −12; 6 mm sphere), and the previously mentioned ROIs. Within each ROI, the average BOLD time series was calculated across all voxels and bivariate temporal correlations, i.e. functional connectivity measures, were computed. For task-related changes in functional connectivity, bivariate regression coefficients were calculated.

CONN’s default setting is to apply Fisher’s Z-transformation, producing ROI-to-ROI correlation matrices. For each participant, Z-transformed values were extracted from the RSATL-ROI pairs for self-blame, other-blame and fixation conditions and imported into SPSS version 26.0 (IBM Corp., Armonk, NY). Other contrasts were created to examine connectivity in the context of self-blaming emotions (self-agency vs fixation), other-blaming emotions (other-agency vs fixation) and the subtraction-based difference between self- and other-blaming emotions for each split half.

Using the imported values for the BOLD-, PPI- and CONN-models comparing response to self-blaming, other-blaming and self- vs other-blaming emotions, ICCs and their 95% confidence intervals were calculated for each ROI using SPSS. These were based on an absolute agreement, two-way random-effects model, which is equivalent to the second ICC (ICC(2,1)) as defined by Shrout and Fleiss (Shrout and Fleiss, 1979). In the context of split-half reliability, it shows to what extent the split halves reflect the same scores for the same subjects (Chen et al., 2018). Generally, ICCs < 0.4 are considered “poor”, 0.4–0.59 as “fair”, 0.6–0.74 as “good” and ≥ 0.75 as “excellent” (Chen et al., 2018, Cicchetti, 2001).

In addition to exploring reliability at a ROI-level, ICCs were calculated at a voxel-level using the MATLAB-based ICC toolbox (Caceres et al., 2009). Even if activated volumes are the same across two sessions, this does not inform as to whether all voxels remained consistently activated, which could be problematic when averaging over potentially functionally heterogenous regions-of-interest (Bennett and Miller, 2010, Caceres et al., 2009, Tarhan and Konkle, 2020). Median ICC estimates and intra-voxel ICC estimates were obtained for the same set of regions-of-interest as mentioned above, and voxel-wise ICC maps were generated using the third ICC (ICC(3,1)) as defined by Shrout and Fleiss (Shrout and Fleiss, 1979), which focuses on consistency, i.e. the extent to which split half values match after accounting for potential systematic differences (Chen et al., 2018). However, if systematic differences are negligible, then ICC(2,1) and ICC(3,1) will be similar (Chen et al., 2018).

#### 2.4.2 fMRI analysis: reliability and reproducibility

Using the full dataset, we investigated the trade-off between split-half reliability and reproducibility of findings between models. We therefore selected the models with optimised split-half reliability when compared against the previously published models and investigated whether these more reliable models could replicate the results of the previously published models: 1) the BOLD model without time and dispersion derivatives (duration 0 s), 2) the SPM PPI based on the first-level BOLD models without time and dispersion derivatives (duration 0 s) and 3) the CONN gPPI model at the second-level (between-subject).

We followed the random-effect BOLD-analysis approach outlined in (Lythe et al., 2020), while we used the same set-up previously outlined in (Lythe et al., 2015) to investigate between-group PPI differences on the contrast of self-blaming vs other-blaming emotions. However, unlike our original PPI paper (Lythe et al., 2015), we applied more stringent cluster-forming uncorrected thresholds for cluster-level correction for multiple comparisons (uncorrected p-values of 0.001). We used an anterior subgenual cingulate cortex (SCC) ROI as described above (MNI coordinates: x = −4, y = 23, z = −5; 6 mm sphere) for voxel-level correction at *p* = .05 and to extract cluster average regression coefficients. For further details, please see Supplementary Methods.

For the gPPI model in CONN, seed-to-voxel analyses were conducted, exploring the effective connectivity between the seed ROI and all other voxels in the brain. Using random-effect analyses, connectivity measures were explored for main effect of group (stable MDD vs recurring MDD), main effect of condition (self- vs other-blame) and interaction effect of group by condition. Results were thresholded at *p* = .001 (uncorrected voxel-level) and corrected for Family-Wise Error (FWE) at cluster-level or voxel-level at *p* = .05 over the previously used a priori SCC ROI (MNI coordinates x = −4, y = 23, z = −5; 6 mm sphere) and the whole brain, respectively.

## 3 Results

### 3.1 Split-half reliability

**Supplementary figure 3:**
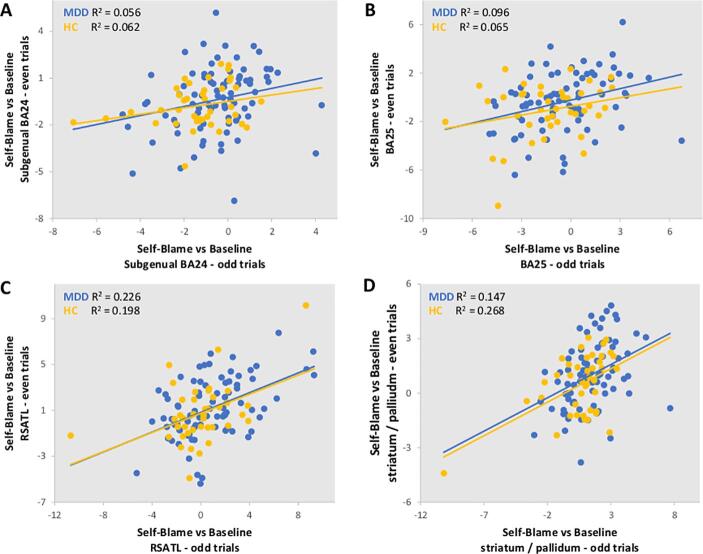


**Supplementary figure 4:**
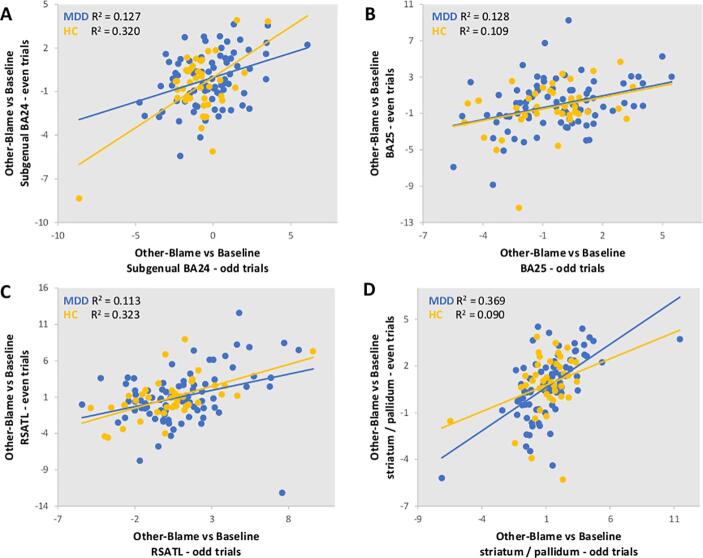


Across ROIs, the BOLD models which included time and dispersion derivates showed poor reliability: most of the ICCs did not exceed 0.4 (Table 2). Some of the measures even showed negative reliability, implying that the split halves showed as much variance as any two randomly selected halves and suggesting inconsistent activation (Nord et al., 2017, Cronbach and Hartmann, 1954). Internal consistency did not differ between remitted MDD and controls (Supplementary Figure 3 and Supplementary Figure 4).

**Table 2:** ICCs for fMRI BOLD measures.

	RSATL	Subgenual (BA24)	Subgenual (BA25)	Striatum / pallidum
TD, d = 0 sec				
SA vs fix	−0.080 (−0.577 to 0.261)	0.184 (−0.194 to 0.442)	0.283 (−0.049 to 0.510)	0.014 (−0.443 to 0.326)
OA vs fix	0.235 (−0.110 to 0.474)	−0.069 (−0.533 to 0.259)	0.352 (0.058 to 0.555)	0.083 (−0.340 to 0.372)
SA vs OA	−0.049 (−0.525 to 0.280)	0.011 (−0.450 to 0.325)	0.166 (−0.222 to 0.431)	−0.095 (−0.597 to 0.250)
TD, d = 5 sec				
SA vs fix	−0.037 (−0.515 to 0.290)	0.013 (−0.443 to 0.324)	0.052 (−0.385 to 0.351)	−0.047 (−0.530 to 0.283)
OA vs fix	0.064 (−0.372 to 0.361)	0.249 (−0.091 to 0.484)	0.361 (0.070 to 0.561)	0.534* (0.319 to 0.681)
SA vs OA	−0.028 (−0.502 to 0.296)	0.003 (−0.454 to 0.316)	0.026 (−0.42 to 0.332)	−0.042 (−0.523 to 0.287)
No TD, d = 0 sec				
SA vs fix	0.650** (0.490 to 0.760)	0.395 (0.116 to 0.587)	0.450* (0.195 to 0.624)	0.583* (0.393 to 0.714)
OA vs fix	0.551* (0.343 to 0.693)	0.636** (0.468 to 0.751)	0.527* (0.308 to 0.676)	0.671** (0.517 to 0.775)
SA vs OA	0.003 (−0.460 to 0.318)	0.340 (0.032 to 0.549)	−0.016 (−0.491 to 0.306)	0.356 (0.059 to 0.560)
No TD, d = 2 sec				
SA vs fix	0.673** (0.523 to 0.776)	0.434* (0.172 to 0.613)	0.408* (0.133 to 0.596)	0.469* (0.227 to 0.635)
OA vs fix	0.639** (0.473 to 0.753)	0.549* (0.340 to 0.691)	0.420* (0.151 to 0.603)	0.669** (0.514 to 0.775)
SA vs OA	0.070 (−0.354 to 0.362)	0.258 (−0.084 to 0.492)	−0.123 (−0.647 to 0.233)	0.417* (0.148 to 0.601)
No TD, d = 5 sec				
SA vs fix	0.609** (0.430 to 0.732)	0.365 (0.071 to 0.566)	0.369 (0.075 to 0.569)	0.411* (0.142 to 0.596)
OA vs fix	0.673** (0.524 to 0.776)	0.362 (0.070 to 0.563)	0.249 (−0.098 to 0.486)	0.577* (0.384 to 0.709)
SA vs OA	0.150 (−0.228 to 0.414)	0.128 (−0.268 to 0.401)	0.084 (−0.339 to 0.374)	0.387 (0.103 to 0.581)
ICC (95% confidence interval, lower to upper bound), n = 109. ICC = intraclass correlation coefficient; BOLD = blood oxygen level-dependent; RSATL = right superior anterior temporal lobe; BA = Brodmann Area; TD = time and dispersion derivatives; SA = self-agency condition; OA = other-agency condition; fix = fixation condition; d = duration. * = fair reliability, ** = good reliability.				

In contrast, BOLD models without time and dispersion derivates resulted in more consistent activation in both self- and other-blame conditions. With a modelled event duration of 0 s, the RSATL showed good reliability for self-blaming emotions, while subgenual BA24 and the striatum / pallidum showed good reliability for other-blaming emotions. In addition, the RSATL displayed fair reliability for other-blaming emotions, while the striatum / pallidum showed fair reliability for self-blaming emotions, and BA25 showed fair reliability for both self-blaming and other-blaming emotions. The BOLD model based on a temporal split, i.e. first and second half of the task as opposed to odd and even trials, showed fair reliability for other-blaming emotions in the RSATL and striatum / pallidum (Supplementary Table 1). Across different BOLD models and ROIs, however, the complex contrast of self- vs other-blaming emotions showed poor reliability (Table 2).

With regard to the internal consistency of effective connectivity, the SPM PPI model showed poor reliability across ROIs and conditions (Table 3). In fact, all measures were well below the “fair” threshold for ICCs and most of the reliability measures were negative. The CONN gPPI model performed marginally better. However, contrasts still showed poor reliability, which is further illustrated by the discrepancy in connectivity values derived from the odd- vs even-numbered trials (Supplementary Table 2). Interestingly, self-blaming and other-blaming emotions on their own, i.e. when not assessed relative to the low-level visual fixation condition, showed fair reliability for subgenual BA24 and BA25, while striatum / pallidum showed poor reliability. A similar trend was observed for the functional connectivity measures: contrasts showed poor reliability across ROIs, while the simple self- and other-blame conditions displayed fair to good reliability.

**Table 3:** ICCs for fMRI functional and effective connectivity measures (PPI, bivariate correlation, gPPI).

	Subgenual (BA24)	Subgenual (BA25)	Striatum / pallidum
SPM, PPI based on BOLD models with time and dispersion ^a^			
SA vs fix	−0.250 (−0.839 to 0.149)	−0.168 (−0.712 to 0.202)	−0.363 (−1.002 to 0.071)
OA vs fix	−0.481 (−1.167 to −0.012)	−0.050 (−0.543 to 0.285)	−0.220 (−0.795 to 0.169)
SA vs OA	−0.152 (−0.692 to 0.215)	−0.028 (−0.512 to 0.299)	−0.222 (−0.797 to 0.168)
SPM, PPI based on BOLD models without time and dispersion			
SA vs fix	0.029 (−0.419 to 0.336)	0.022 (−0.430 to 0.331)	−0.005 (−0.469 to 0.312)
OA vs fix	−0.435 (−1.107 to 0.021)	−0.011 (−0.481 to 0.309)	−0.161 (−0.705 to 0.208)
SA vs OA	−0.082 (−0.586 to 0.261)	−0.041 (−0.521 to 0.288)	−0.020 (−0.491 to 0.303)
CONN, correlation			
SA	0.562* (0.357 to 0.701)	0.669** (0.516 to 0.774)	0.682** (0.534 to 0.783)
OA	0.698** (0.558 to 0.793)	0.603** (0.419 to 0.729)	0.688** (0.543 to 0.787)
SA vs fix	−0.178 (−0.733 to 0.198)	−0.088 (−0.591 to 0.256)	−0.545 (−1.273 to −0.052)
OA vs fix	0.217 (−0.149 to 0.466)	−0.223 (−0.799 to 0.168)	−0.298 (−0.897 to 0.112)
SA vs OA	0.036 (−0.415 to 0.343)	−0.290 (−0.890 to 0.119)	−0.298 (−0.908 to 0.115)
CONN, gPPI			
SA	0.422* (0.153 to 0.606)	0.555* (0.348 to 0.697)	0.222 (−0.140 to 0.469)
OA	0.504* (0.276 to 0.661)	0.439* (0.180 to 0.617)	0.374 (0.088 to 0.571)
SA vs fix	−0.062 (−0.559 to 0.276)	0.041 (−0.407 to 0.346)	−0.269 (−0.864 to 0.135)
OA vs fix	0.297 (−0.032 to 0.520)	−0.172 (−0.724 to 0.202)	0.148 (−0.243 to 0.416)
SA vs OA	0.371 (0.080 to 0.570)	0.100 (−0.319 to 0.385)	0.005 (−0.460 to 0.322)
^a^ one participant was excluded from this analysis due to data issues; the final sample is n = 120 rather than n = 121 for all the other models. ICC (95% confidence interval, lower to upper bound). ICC = intraclass correlation coefficient; BOLD = blood oxygen level-dependent; PPI = psychophysiological interaction; gPPI = generalised psychophysiological interaction; MDD = major depressive disorder; HC = healthy control; SA = self-agency condition; OA = other-agency condition; fix = fixation condition. * = fair reliability, ** = good reliability.			

**Fig. 1:**
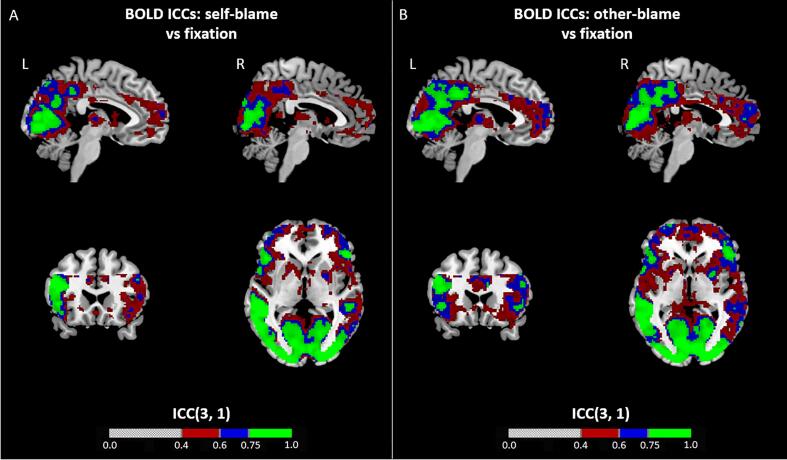
Whole-brain voxel-wise ICC maps for other- and self-blame condition BOLD signal. Whole-brain voxel-wise ICC maps for self-blame vs fixation (panel A) and other-blame vs fixation (panel B) contrasts overlaid on MRIcron’s “ch2better” template (Rorden and Brett, 2000). Contrasts were created using the model without time and dispersion derivatives, with a duration of 0 s. ICCs are displayed according to reliability range, where red = fair (0.4 – 0.59), blue = good (0.6 – 0.74), green = excellent (>0.75). MNI coordinates for each slice: left (L) sagittal (x = −5), right (R) sagittal (x = 4), axial (z = −1) and coronal (y = 22). BOLD = blood oxygen level-dependent; ICC = intraclass correlation coefficient; MNI = Montreal Neurological Institute. (For interpretation of the references to colour in this figure legend, the reader is referred to the web version of this article.)

At a voxel-level, other-blaming emotions resulted in more consistent BOLD activation compared to self-blaming emotions, particularly in the frontal cortex (see Fig. 1). In both conditions, brain areas associated with visual processing showed excellent reliability, while the specific regions-of-interest we chose displayed fair intra-voxel reliability. The voxel-wise reliability of the ROIs was further quantified using median ICC and intra-voxel ICC measures (see Table 4). Unlike the ICC maps, the reliability measures showed poor reliability for the ROIs, except for the RSATL and striatum / pallidum. However, a similar trend was observed for other-blaming emotions resulting in more consistent activation relative to self-blaming emotions. Generally, the subgenual regions displayed the lowest reliability in both the ICC maps as well as the ICC measures.

**Table 4:** Voxel-wise reliability measures for self- and other-blame condition BOLD signal in *a priori* ROIs from ICC toolbox.

ROI	median ICC (SE)		intra-voxel ICC (SE)	
	d = 0 sec	d = 2 sec	d = 0 sec	d = 2 sec
*Subgenual BA24:* SA vs fix	0.27 (0.02)	0.31 (0.02)	0.35 (0.05)	0.36 (0.05)
*Subgenual BA24:* OA vs fix	0.35 (0.01)	0.33 (0.01)	0.23 (0.05)	0.26 (0.06)
*BA25*: SA vs fix	0.25 (0.01)	0.23 (0.01)	0.30 (0.05)	0.32 (0.04)
*BA25:* OA vs fix	0.29 (0.01)	0.22 (0.01)	0.31 (0.05)	0.33 (0.06)
*RSATL:* SA vs fix	0.44* (0.01)	0.47* (0.01)	0.35 (0.04)	0.37 (0.05)
*RSATL:* OA vs fix	0.35 (0.01)	0.40* (0.01)	0.41* (0.03)	0.46* (0.07)
*striatum / pallidum:* SA vs fix	0.35 (0.00)	0.30 (0.00)	0.38 (0.03)	0.37 (0.02)
*striatum / pallidum:* OA vs fix	0.38 (0.00)	0.36 (0.00)	0.40* (0.03)	0.49* (0.05)
BOLD = blood oxygen level-dependent; ROI = region-of-interest; ICC = intraclass correlation coefficient; SE = standard error; d = duration; SA = self-agency condition; OA = other-agency condition; fix = fixation condition; RSATL = right superior anterior temporal lobe. * = fair reliability.				

### 3.2 Reproducibility

#### 3.2.1 Reproducibility: BOLD model

**Fig. 2:**
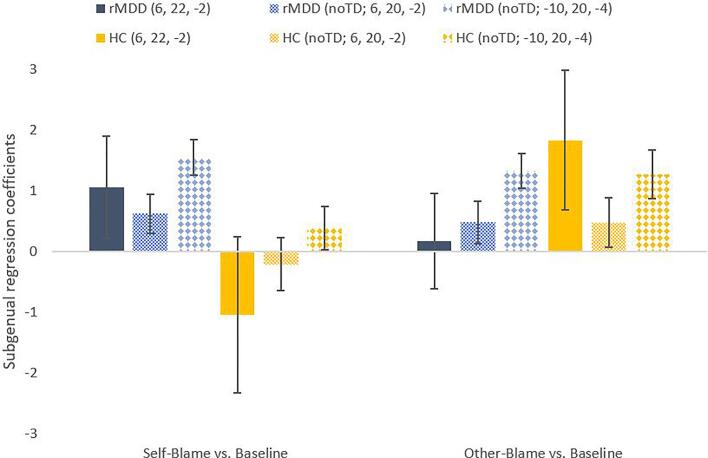
Extracted regression coefficient cluster averages and standard errors for the right and left subgenual cingulate regions as identified by BOLD models, showing the interaction effect between group (rMDD [n = 70] vs control [n = 39]) and condition (self- vs other-blaming). The bar charts show the extracted regression coefficient cluster averages and standard errors for the right subgenual cingulate region (BA24) as identified by (Lythe et al., 2020) (MNI: x = 6, y = 22, z = −2), the right subgenual cingulate region as extracted for the model with no time and dispersion derivatives (MNI: x  = 6, y = 20, z = −2), and the left subgenual cingulate region (BA24) as extracted for the model with no time and dispersion derivatives (MNI: x = −10, y = 20, z = −4). BA = Brodmann Area; BOLD = blood oxygen level-dependent; MNI = Montreal Neurological Institute; rMDD = remitted major depressive disorder; HC = healthy control; TD = time and dispersion derivatives.

Next, we sought to replicate the findings as reported in (Lythe et al., 2020), which showed an interaction between group (remitted MDD vs control) and condition (self- vs other-blaming emotions) in the right SCC (Table 5). This was due to higher SCC signal for self-blame in remitted MDD and higher other-blame-selective activation in control participants (Fig. 2).

**Table 5:** Factorial models for fMRI activation in remitted MDD and control group.

*MNI peak coordinates*								
Hemisphere	Region	Cluster size	Brodmann Area	x	y	z	F-value	Voxel-based FWE-corrected *p* value
Group × condition interaction effect: Lythe et al., 2020								
right	Anterior subgenual cingulate cortex	11	24	6	22	−2	9.46	.004^a^
Group × condition interaction effect: reproduction								
right	Anterior subgenual cingulate cortex	1	24	6	20	−2	8.34	.014^a^
Group × condition interaction effect: no time and dispersion derivatives modelled								
left	Anterior subgenual cingulate cortex	8	24	−10	20	−4	9.78	.06^b^
^a^ Using *a priori* subgenual cingulate region of interest (6 mm sphere, MNI: x = −4, y = 23, z = −5, (Green et al., 2012)) for multiple comparison correction. There were no main effects of group or condition in this region. No voxels survived voxel-based FWE-correction over the whole brain at *p* = .05 for main effects or interactions. ^b^ Using *a priori* subgenual cingulate region of interest (6 mm sphere, MNI: x = −4, y = 23, z = −5, (Green et al., 2012)) for multiple comparison correction. There was no main effect of group in this region, but there was a main effect for condition. No voxels survived voxel-based FWE-correction over the whole brain at *p* = .05 for main effects or interactions. MDD = major depressive disorder; FWE = family-wise error; MNI = Montreal Neurological Institute

Using the same modelling approach (Lythe et al., 2020), but with the minor discrepancies previously noted corrected, we found similar results (Table 5). Interestingly, using the approach that yielded the most reliable measures, i.e. modelling without time and dispersion derivatives, we observed a significant interaction effect between group (remitted MDD vs control) and condition (self- vs other-blaming emotions) in the left SCC rather than the right SCC (Table 5). As in (Lythe et al., 2020), this interaction effect was driven by a higher SCC signal for self-blame in the remitted MDD group relative to other-blame, while the control group showed a lower SCC signal for self-blame relative to other-blame (see Supplementary Results).

#### 3.2.2 Reproducibility: PPI models

We sought to replicate the SPM PPI findings as reported in (Lythe et al., 2015). Compared with stable MDD participants, those with a recurring major episode exhibited a hyper-connectivity with the RSATL seed region for self- vs other-blame. More specifically, this hyper-connectivity was found in the posterior subgenual cortex and adjacent septal region, the right ventrolateral putamen (extending into the claustrum) and the right temporoparietal junction. Using the more stringent cluster-forming threshold of *p* = .001 (uncorrected voxel-level), only the right ventrolateral putamen (extending into the claustrum) remains. We were able to reproduce these findings using the same modelling approach (again with the minor discrepancies corrected) and using the modelling approach without time and dispersion derivatives (Table 6). For the full independent re-analysis, please see Supplementary Results.

**Table 6:** PPI effects for self- vs other-blaming emotions using SPM software.

	*MNI peak coordinates*						
	Anatomical region	Cluster size	x	y	z	t-statistic	FWE-corrected *p* value
*Lythe et al.* (Lythe et al., 2015)*:*							
Recurring episode MDD > stable remission MDD							
	Ventrolateral putamen and claustrum	611	32	8	−2	4.88	.01^a^
	Posterior SCSR (BA25)	56	2	14	−6	3.59	.05^b^
*Replication:*							
Recurring episode MDD > stable remission MDD							
	Ventrolateral putamen and claustrum	620	32	8	−2	4.78	.007^a^
	Posterior SCSR (BA25)	76	2	14	−6	3.63	.04^b^
*No time and dispersion derivatives modelled:*							
Recurring episode MDD > stable remission MDD							
	Ventrolateral putamen and claustrum	620	32	8	−2	4.79	.007^a^
	Posterior SCSR (BA25)	78	2	14	−6	3.63	.04^b^

RSATL PPI effects for the recurring episode vs. the stable remission MDD group (self- vs other-blame emotions) as in the previous paper [Lythe et al. (Lythe et al., 2015), and its replication, as well as a model with no time and dispersion derivatives were compared. ^a^ Region surviving inclusive masking at uncorrected *p* = .001, with cluster-level FWE correction over the whole brain. ^b^ Region surviving voxel-based FWE correction over the *a priori* SCSR using small-volume correction. BA = Brodmann Area; PPI = psychophysiological interaction; RSATL = right superior anterior temporal lobe; SCSR = subgenual cingulate cortex and adjacent septal region; MDD = major depressive disorder; FWE = family-wise error; MNI = Montreal Neurological Institute.

In addition, we investigated the dataset using the CONN gPPI approach, which had shown higher split-half reliability than SPM PPI. The dataset had not been explored using CONN before, so this offered a chance to investigate whether we could obtain similar findings as when using SPM PPI and whether the gPPI approach would reveal any other differences in connectivity patterns between participants with recurring MDD and participants with stable MDD. Unlike the SPM PPI model, however, we did not observe any significant differences in connectivity between recurring MDD and stable MDD groups using the CONN gPPI approach. Moreover, we did not find a main effect of condition (self- vs other-blame) or an interaction effect of group by condition.

## 4 Discussion

In recent years, reliability of fMRI results has been given more and more attention, but there is little consensus whether fMRI captures reliable measures of neural activity: some suggest fair reliability (Bennett and Miller, 2010), while others point to poor reliability (Elliott et al., 2020). Paradigm choices (Elliott et al., 2020, Hedge et al., 2018, Bennett and Miller, 2013), scanners (Gorgolewski et al., 2013, Friedman et al., 2006) and modelling approaches (Fournier et al., 2014) add to the variability of the findings across studies, and even the same dataset can give different results based on the ICC chosen (Müller and Büttner, 1994). However, for fMRI measures to have any potential clinical application, it is key to obtain consistent findings and to be aware of potential limitations.

Here, we probed the reliability of our blame biases paradigm in a relatively large sample of MDD and healthy control participants. Using different modelling approaches, we showed fair reliability for simple fMRI measures related to self-blame, but poor reliability for more complex measures. Our findings corroborate previous reports that reliability appears to diminish with increasing model complexity, especially when using contrasts or difference scores (Hedge et al., 2018, Gorgolewski et al., 2013, Fröhner et al., 2019, Heckendorf et al., 2019). Infantolino and colleagues showed that, when two conditions are highly correlated in an individual, subtraction-based difference scores will invariably remove shared variance which is relevant to the internal consistency of the task (Infantolino et al., 2018). It is plausible that the self- and other-agency conditions might be correlated and share reliable variance to a degree, resulting in poor reliability estimates for the self- vs other-agency contrast.

Moreover, measures of functional and effective connectivity are inherently more complex than BOLD activation, which is reflected in the poor reliability estimates for our PPI models. PPI effects are relatively noisy measures, especially compared to main effects, as they contain the noise of both psychological and physiological factors in its interaction term, potentially accumulating the error term (Di and Biswal, 2017). Even though our gPPI model displayed fair reliability for subgenual-ATL connectivity in both self- and other-blame conditions, this measure did not account for baseline activity and was unable to differentiate between clinical groups. In contrast, Nord and colleagues reported more reliable estimates for PPI effects compared to BOLD response (Nord et al., 2019) – the opposite pattern to our finding. As they used emotional faces tasks and focussed on the amygdala, this may indicate that reliability estimates are task- and region-dependent.

Notably, we observed fair to good reliability for both self- and other-blame conditions in our BOLD models without derivatives, while our BOLD models with time and dispersion derivatives displayed poor reliability. We expected the opposite: including derivatives has been shown to improve specificity and sensitivity of the HRF by accounting for as much variability as possible (Handwerker et al., 2004, Lindquist et al., 2009), which should lead to better estimates of the BOLD signal, and therefore larger ICCs, compared to models without derivatives. One possible explanation could be that by capturing variance related to temporal and spatial factors, variance relevant to its internal consistency is inadvertently removed. However, it should be noted that the comparison between models with and without derivatives is not straightforward – time and dispersion derivatives bias the magnitude of amplitude, affecting its reliability estimates. Ideally, models using derivatives should include a weighted combination of time and dispersion derivatives (Calhoun et al., 2004), but this is uncommon practice in the field.

The difference in internal consistency displayed by the various modelling approaches did not lead to major discrepancies at the group-level, except for a change in lateralisation of the peak of the subgenual region BOLD activation. Our observation that it is possible to have robust activation at the group-level despite weak consistency at the individual level is in line with previous reports (Di and Biswal, 2017, Infantolino et al., 2018, McDermott et al., 2020, Smith et al., 2016). It reflects a trade-off between within- and between-subject variability, which are the same elements that determine the ICC. Most fMRI studies in psychiatric disorders rely on between-subject differences, which are driven by high variability between individuals (Hedge et al., 2018). On the other hand, fMRI studies in healthy control populations typically aim to uncover consistent associations between brain response and certain functions across subjects, which requires low variability between individuals, thus lowering their potential to differentiate between individuals (Hedge et al., 2018). There is thus a balance to be struck when selecting measures as potential biomarkers, so that they can differentiate between individuals, but are also based on relatively consistent neural activations within individuals.

Somewhat counterintuitive, it is possible for a decline in reliability to result in an increase in validity (Feldt, 1997), which seems to be reflected in our findings. Irrelevant factors to the fMRI measure itself, for instance physiological noise and motion, tend to exhibit high reliability (Noble et al., 2019). As such, a task can be reliable in not measuring any meaningful activation (Gorgolewski et al., 2013). There are many approaches available to account for these artifacts and improve validity, each resulting in considerable variation in activation strength, location and extent based on pre-processing and model estimation parameters chosen (Carp, 2012), which in turn is likely to affect reliability estimates. However, stability of the underlying neural signal has been shown to contribute more to the consistency of BOLD response than physiological noise (Lipp et al., 2014).

It is important to recognise that many of our ICCs were negative, especially for the models with time and dispersion derivatives, pointing to inconsistent activation (Nord et al., 2017, Cronbach and Hartmann, 1954). However, it is not uncommon for neuroimaging analyses to yield negative ICC values, which can occur when the variance of the total score is less than the sum of between- and within-subject variance or when there is unequal covariance among the split-halves (Chen et al., 2018, Cronbach and Hartmann, 1954, Parsons et al., 2019). Moreover, split-half reliability estimates have the potential to vary depending on which trials are included (Parsons et al., 2019, Warrens, 2014). However, in our case, the odd-even split ICC values were similar to the first-second half split ICC values.

Even though most of the neural measures display poor ICC values, it does not mean that our paradigm is inherently unreliable. The low values could be a reflection of low between-subject variability rather than high variability in the measure (Hedge et al., 2018), or insufficient modelling of confounding effects (Chen et al., 2018). Moreover, the BOLD signal itself is intrinsically variable, implying that activation itself could be reliable, but that a low ICC reflects the variance in the amplitude of the activation (Raemaekers et al., 2012). In addition, we focussed on the reliability within a set of predefined ROIs, which might be misleading as activation does not imply reliability per se (Caceres et al., 2009, Gorgolewski et al., 2013).

Lastly, we reported on the internal consistency of our self-blame-related fMRI measures, which does not necessarily convey information about its test-retest reliability. In fact, it is possible to have good internal consistency, but poor test-retest reliability and vice versa (Hajcak et al., 2017). It is important to reiterate that test-retest reliability examines the temporal stability of a measure, which is important if the prospective biomarker aims to capture trait-like brain processes (Kragel et al., 2021). However, without evaluating internal consistency, it is difficult to unravel the interplay between trait and state features and whether the measure truly captures what it intends to capture. Thus, it would be interesting to see how our self-blame-related fMRI measures perform over repeated time points, which could also provide insight about the intrinsic variability of self-blaming biases in MDD.

## 5 Conclusion

Internal consistency of self-blame-related fMRI measures was probed using different modelling approaches, which showed that relatively simple measures had better reliability compared with more complex contrasts. While simple BOLD contrasts had fair reliability, previously employed SPM PPI models had poor reliability and simple CONN toolbox connectivity measures lacked clinical validity (i.e. predictive of recurrence risk). This calls for the development of functional connectivity measures that strike a better balance between reliability and validity for future clinical applications, for which individual, not group-level, results are paramount.

### CRediT authorship contribution statement

**Diede Fennema:** Conceptualization, Methodology, Formal analysis, Writing – original draft, Visualization. **Owen O'Daly:** Conceptualization, Methodology, Writing – review & editing. **Gareth J. Barker:** Conceptualization, Methodology, Writing – review & editing. **Jorge Moll:** Conceptualization, Writing – review & editing. **Roland Zahn:** Conceptualization, Methodology, Data curation, Writing – review & editing, Supervision, Funding acquisition.

## Declaration of Competing Interest

The authors declare that they have no known competing financial interests or personal relationships that could have appeared to influence the work reported in this paper.

## Figures and Tables

**Supplementary figure 5:**