# Supplementary material for: Internal reliability of blame-related functional MRI measures in major depressive disorder
Source: Neuroimage Clin. 2021 Nov 28;32:102901. doi: 10.1016/j.nicl.2021.102901 (PMC8640114; doi:10.1016/j.nicl.2021.102901)
Supplement: Supplementary data 1 [file mmc1.docx]

**SUPPLEMENTAL INFORMATION**

**Internal reliability of blame-related functional MRI measures in major depressive disorder**

Diede Fennema^1^, Owen O’Daly^2^, Gareth J. Barker^2^, Jorge Moll^3^, & Roland Zahn^1,3,4,*^

^1^ Department of Psychological Medicine, Institute of Psychiatry, Psychology & Neuroscience, Centre for Affective Disorders, King’s College London, London, SE5 8AZ, UK

^2^ Department of Neuroimaging, Institute of Psychiatry, Psychology & Neuroscience, King’s College London, London

^3^ Cognitive and Behavioural Neuroscience Unit, D’Or Institute for Research and Education (IDOR), 22280-080, Rio de Janeiro, RJ, Brazil

^4^ National Service for Affective Disorders, South London and Maudsley NHS Foundation Trust, London, SE5 8AZ

* Corresponding author

Dr Roland Zahn (see address above)

E-mail: roland.zahn@kcl.ac.uk

Phone: 0044-(0)20 7848 0348

Fax: 0044-(0)20 7848 0298

**Short title:**

*Keywords:* fMRI; split-half reliability; internal consistency; intraclass coefficient correlation; self-blame; depression

# 1. Supplementary Methods

## 1.1. Image acquisition details

As previously described [1], an fMRI protocol optimised for detection of ventral brain regions was used. T2*-weighted echo-planar images (3 runs of 405 volumes with 5 dummy scans) were acquired on an MRI scanner (3T Achieva, Philips) with an 8-channel head coil, 3mm section thickness, ascending continuous acquisition parallel to the anterior to posterior commissural line, 35-40 slices depending on the participant's head size, repetition time=2000 milliseconds, echo time=20.5 milliseconds, field of view=220x220x120mm, acquisition matrix=80 x 80 voxels, reconstructed voxel size=2.29x2.29x3mm, and sensitivity encoding factor=2, enabling dynamic stabilisation to correct for signal drift.

T1-weighted, magnetization-prepared, rapid-acquisition gradient-echo structural images were obtained using 160 axial slices; 9mm slice thickness; repetition time=8.4ms; echo time=3.9ms; field of view=240x191x144mm; acquisition matrix=256x163 voxels; reconstructed voxel size=.94 x.94x.9mm; flip angle: 8°.

## 1.2. Modelling duration (0, 2 or 5 seconds)

In theory, the fMRI paradigm we used is likely to involve the following steps: i) read the sentence; ii) understand the sentence; iii) process the agency-context; iv) pick an example; v) evaluate the emotional relevance, and vi) press the button. In addition to the early, implicit processing (steps i and ii), the focus is on the parallel processing taking place in the subgenual and its relevant circuits (steps iii, iv, and v).

Previous unpublished work by Zahn and colleagues explored the timings of the various processes using an electroencephalography (EEG) version of the value-related moral sentiment task, as EEG is known for its temporal resolution [4]. This EEG paradigm differed slightly from the fMRI one in that it only showed the participant’s name, a social concept and the best friend’s name, resulting in a shorter reading time. In contrast, the fMRI paradigm added a word to form a sentence, possibly “delaying” the processing of the agency-context compared to the EEG paradigm.

Within the field of EEG, there is strong evidence that there is a negative peak at 400ms for semantic priming [5], which was also observed in this EEG version. As the fMRI paradigm contained four words, it might take around 1600ms for the last word to be processed (four times N400). Furthermore, the average response time was around 2350ms, with no difference between major depressive disorder (MDD) and healthy controls [6]. The last 400ms or so would be likely to reflect the decision-making and pressing the button (step vi), which is not relevant to the task itself.

Therefore, it was opted to model trial-length using three different durations (0, 2 and 5 seconds). In addition to modelling the various durations, one could also consider including the trial-by-trial response time as a parametric variable to help explain trial-by-trial variability. However, we are assuming that our main region-of-interest, the subgenual region, is unaffected by response time. Based on the EEG version and unpublished work, the more complex neural responses appear not to be strictly time-locked in keeping with a parallel distributed processing framework.

## 1.3. Setting up fMRI analysis: BOLD analysis

In line with Lythe and colleagues [6], a random-effect BOLD-analysis was performed in SPM using a factorial model with two factors: group (remitted MDD vs control) and condition (self- vs other-blame). Within the model set-up, no independence was assumed for condition as both self- and other-blame were measured within the same participant. F-contrasts for main effects of group, condition and their interaction were thresholded at *p* = .005 (uncorrected voxel-level) and corrected for Family-Wise Error (FWE) at the voxel-level at *p* = .05 over an *a priori* anterior subgenual cingulate cortex (SCC) region-of-interest (ROI) (MNI coordinates x = -4, y = 23, z = -5; 6mm sphere) and the volume of the whole brain. The SCC cluster average regression coefficients for each condition (self-blame vs fixation and other-blame vs fixation) were extracted for each participant using MarsBar [7] to explore the interaction effects.

## 1.4. Setting up fMRI analysis: PPI analysis

In line with Lythe and colleagues [8], between-group psychophysiological interaction (PPI) differences on the contrast of self-blaming vs other-blaming emotions were tested in a two-sample t-test using SPM: stable MDD vs recurring MDD. Since our original PPI paper [8], the generally accepted best practice within the field has changed to using more stringent cluster-forming uncorrected thresholds for cluster-level correction for multiple comparisons (uncorrected *p*-values of .001) to address concerns raised by Eklund and colleagues [9]. Therefore, while PPI results were thresholded at *p* = .001 (uncorrected voxel-level) as is currently recommended [10], we also used the less stringent cluster-forming threshold of *p* = .005 (uncorrected voxel-level) to allow for direct comparison with the original findings [8]. Results were corrected for FWE at cluster-level or voxel-level at *p* = .05 over the previously mentioned *a priori* SCC ROI (MNI coordinates: x = -4, y = 23, z = -5; 6 mm sphere) and the whole brain. The cluster average regression coefficients from the comparisons between the recurring and stable MDD groups were extracted for each participant using MarsBar [7] to explore the interaction effects.

# 2. Supplementary Results

## 2.1. Reliability and reproducibility BOLD model

The interaction effect in the left SCC was confirmed by examining the extracted cluster averages for this region (*F*(1,107) = 8.28, *p* = .005), with no main effect of agency (*F*(1,107) = 3.03, *p* = .08) or group (*F*(1,107) = 1.95, *p* = .17). Like [6], this interaction effect was driven by a higher SCC signal for self-blame in the remitted MDD group (M = 1.54, SD = 2.46) relative to other-blame (M = 1.32, SD = 2.41), resulting in a positive difference for self-blame vs other-blame (M = 0.22, SE = 0.24, t = 0.93, df = 69, *p* = 0.36). Similarly to [6], the control group showed a lower SCC signal for self-blame (M = 0.38, SD = 2.20) relative to other-blame (M = 1.26, SD = 2.46), resulting in a negative difference for self- vs other-blame (M = -0.89, SE = 0.29, t = -3.01, df = 38, *p* = .005). There was a significant difference between groups on self-blame vs other-blame differences (mean difference = -1.11, SE = 0.38, t = -2.88, df = 107, *p* = .005), which was identified by the observed interaction effect (Figure 2).

As we were also interested in replicating the original results [6], we explored the extracted cluster averages for the right SCC in the model without time and dispersion derivatives, using the coordinates as identified in the replication model (MNI: x = 6, y = 20, z = -4; see Figure 2). This region did not survive our main analysis and we did not find an interaction of group by condition effect (*F*(1,107) = 3.34, *p* = .07), a main effect of agency (*F*(1,107) = 1.42, *p* = .24) nor a main effect of group (*F*(1,107) = .68, *p* = .41). However, we did observe a similar trend: the remitted MDD group showed a higher SCC signal for self-blame (M = .62, SD = 2.73) relative to other-blame (M = .47, SD = 2.98), while the control group displayed a lower SCC signal for self-blame (M = -.21, SD = 2.69) relative to other-blame (M = .47, SD = 2.54). Moreover, there was a significant difference between the remitted MDD and control groups on self-blame vs other-blame differences (mean difference = -.83, SE = .41, t = -2.05, df = 104.1, *p* = .04).

## 2.2. Independent re-analysis of SPM PPI model

We sought to replicate the SPM PPI findings as reported in [8]. Compared with stable MDD participants, those with a recurring major episode exhibited a hyper-connectivity with the right superior anterior temporal lobe (RSATL) seed region for self- vs other-blame. More specifically, this hyper-connectivity was found in the posterior subgenual cortex and adjacent septal region, the right ventrolateral putamen (extending into the claustrum) and the right temporoparietal junction. Using the less stringent cluster-forming threshold as applied in [8] (uncorrected *p =* .005), we were able to reproduce these findings using the same modelling approach (again with minor discrepancies corrected) and using the modelling approach without time and dispersion derivatives (Supplementary Table 3).

Moreover, participants with recurring MDD displayed higher right superior anterior temporal lobe-subgenual cingulate cortex and adjacent septal region (RSATL-SCSR) connectivity in the self-blaming condition and lower RSATL-SCSR connectivity in the other-blaming condition relative to participants with stable MDD, in line with [8] (Supplementary Figure 5). These RSATL-SCSR connectivity differences were driven by an interaction between emotion (self-blaming vs other-blaming) and group (recurring MDD vs stable MDD).

We observed a similar pattern in the model without time and dispersion derivatives, with the RSATL-SCSR connectivity difference driven by an interaction between emotion and group (*F*(1,54) = 15.8, *p* < .001; Supplementary Figure 5). There was no main effect of emotion (*F*(1,54) = 1.64, *p* = .21) or group (*F*(1,54) = .01, *p* = .94). Like [8], participants with recurring MDD showed higher RSATL-SCSR connectivity in the self-blaming condition (M = .47, SD = 1.01) and lower RSATL-SCSR connectivity in the other-blaming condition (M = -.89, SD = 1.22), resulting in a positive difference for self-blame vs other-blame (M = 1.36, SE = .38, t = 3.62, df = 24, *p* = .001). Participants with stable MDD showed lower RSATL-SCSR connectivity for self-blame (M = -.55, SD = 1.05) and higher RSATL-SCSR connectivity in the other-blaming condition (M = .15, SD = 1.13), resulting in a negative difference for self-blame vs other-blame (M = -.70, SE = .35, t = -1.98, df = 30, *p* = .06). There was a significant difference between groups on self-blame vs other-blame differences (mean difference = 2.06, SE = 0.52, t = 3.98, df = 54, *p <* .001), which was identified by the observed interaction effect.

# Supplementary Figure Legends

**Supplementary Figure 1 | Overview of analyses for probing reliability and validity of self-blame-related fMRI measures**. Internal consistency was calculated for BOLD- and PPI-signals of various *a priori* ROIs, using a split-half approach. To assess the impact of alternative modelling approaches, trial-length was modelled using three durations (0, 2 and 5 seconds), which was convolved with the haemodynamic response function with and without time and dispersion derivates. Validity was evaluated for the previously published BOLD [6] and PPI [8] models and the more reliable model. Based on data collected by [8]. BA = Brodmann Area, RSATL = right superior anterior temporal lobe, TD = time and dispersion derivatives, SPM = Spatial Parametric Mapping, BOLD = blood oxygen-level dependent, PPI = psychophysiological interaction, gPPI = generalised psychophysiological interaction, ROIs = region-of-interest, ICC = intraclass correlation coefficients.

**Supplementary Figure 2 | Overall mean tSNR map across participants for the fMRI paradigm.** Mean tSNR values for each participant (n = 122) were combined into one overall mean tSNR across participants. The tSNR exceeds the minimum threshold of 40 for most regions as proposed by [11]. Based on data collected by [8], displayed using MRIcron [12]. tSNR = temporal signal-to-noise ratio.

**Supplementary Figure 3 | Split-half relationships for self-blaming vs baseline BOLD response (modelling without time and dispersion derivates and duration of 0 seconds).** Internal consistency displayed for subgenual BA24 (**panel A)**, BA25 (**panel B**), RSATL (**panel C**) and pallidum / striatum (**panel D**), which did not differ between rMDD and control group. Additional analysis excluding outliers yielded similar findings, indicating that the outliers are not driving the effects. BOLD = blood oxygen-level dependent; MDD = major depressive disorder; BA = Brodmann Area; RSATL = right superior anterior temporal lobe.

**Supplementary Figure 4 | Split-half relationships for other-blaming vs baseline BOLD response (modelling without time and dispersion derivates and duration of 0 seconds).** Internal consistency displayed for subgenual BA24 (**panel A)**, BA25 (**panel B**), RSATL (**panel C**) and pallidum / striatum (**panel D**), which did not differ between rMDD and control group. Additional analysis excluding outliers yielded similar findings, indicating that the outliers are not driving the effects. BOLD = blood oxygen-level dependent; MDD = major depressive disorder; BA = Brodmann Area; RSATL = right superior anterior temporal lobe.

**Supplementary Figure 5 | Connectivity coefficients for posterior RSATL-SCSR for self-blaming and other-blaming emotions vs baseline, comparing participants with stable MDD and recurring MDD.** The bar charts show the extracted connectivity coefficients and standard errors for the RSATL-SCSR as identified by [8] and the PPI model based on BOLD with no time and dispersion derivatives, for participants with stable major depressive disorder (MDD) [n = 31] and recurring MDD [n = 25]. It shows that the RSATL-SCSR connectivity differences were driven by an interaction between condition (self- vs other-blame) and group (recurring MDD vs stable MDD). BOLD = blood oxygen-level dependent; RSATL = right superior anterior temporal lobe; SCSR = subgenual cingulate cortex and adjacent septal region; TD = time and dispersion derivatives.

**Supplementary Table 1 | ICCs for fMRI BOLD measures using temporal split**

|  | RSATL | Subgenual (BA24) | Subgenual (BA25) | Striatum / pallidum |
| --- | --- | --- | --- | --- |
| No TD, d = 0 sec, first vs second |  |  |  |  |
| SA vs fix | .079  (-.352 to .371) | .395  (.113 to .586) | .276  (-.060 to .505) | .295  (-.031 to .517) |
| OA vs fix | .407*  (.138 to .593) | .359  (.062 to .562) | .295  (-.023 to .516) | .542*  (.334 to .685) |
| SA vs OA | .130  (-.267 to .404) | .435*  (.173 to .614) | .111  (-.299 to .391) | -.359  (-.986 to .070) |
| ICC (95% confidence interval, lower to upper bound), n = 109. ICC = intraclass correlation coefficient; BOLD = blood oxygen-level dependent; RSATL = right superior anterior temporal lobe; BA = Brodmann Area; TD = time and dispersion derivatives; SA = self-agency condition; OA = other-agency condition; fix = fixation condition; d = duration. * = fair reliability, ** = good reliability. | | | | |

**Supplementary Table 2 | RSATL-to-ROI correlation matrix for CONN split-half**

|  | Subgenual (BA24) | | Subgenual (BA25) | | Striatum / pallidum | |
| --- | --- | --- | --- | --- | --- | --- |
|  | **Odd** | **Even** | **Odd** | **Even** | **Odd** | **Even** |
| CONN, weighted GLM: bivariate temporal correlation | | | | | | |
| SA | 0.193 | 0.191 | 0.274 | 0.285 | 0.446 | 0.449 |
| OA | 0.215 | 0.193 | 0.284 | 0.272 | 0.433 | 0.419 |
| SA vs fix | -0.011 | -0.013 | -0.026 | -0.010 | 0.009 | -0.016 |
| OA vs fix | 0.011 | -0.012 | -0.016 | -0.023 | -0.004 | -0.046 |
| SA vs OA | -0.022 | -0.001 | -0.010 | 0.013 | 0.013 | 0.030 |
| CONN, gPPI: bivariate regression coefficients | | | | | | |
| SA | -0.034 | 0.045 | 0.158 | 0.038 | 0.567 | 0.207 |
| OA | 0.543 | -0.068 | 0.309 | -0.246 | 0.446 | -0.142 |
| SA vs fix | -0.361 | -0.131 | -0.447 | -0.361 | 0.183 | -0.381 |
| OA vs fix | 0.216 | -0.245 | -0.296 | -0.645 | 0.061 | -0.730 |
| SA vs OA | -0.577 | 0.114 | -0.151 | 0.284 | 0.122 | 0.349 |
| Fisher transformed connectivity values for each split-half derived from weighted GLM and gPPI models, n = 120. RSATL = right superior anterior temporal lobe; BA = Brodmann Area; SA = self-agency condition; OA = other-agency condition; fix = fixation condition; gPPI = generalised psychophysiological interaction. | | | | | | |

**Supplementary Table 3 | PPI effects for self- vs other-blaming emotions using SPM software, less stringent cluster-forming threshold**

|  | | *MNI peak coordinates* | | | | | | |  | |  |
| --- | --- | --- | --- | --- | --- | --- | --- | --- | --- | --- | --- |
|  | Anatomical region | | Cluster size | | x | y | z | t-statistic | | FWE-corrected *p* value | |
| *Lythe et al. (2015):* | | | | | | | | | | | |
| Recurring episode MDD > stable remission MDD | | | | | | | | | | | |
|  | Ventrolateral putamen and claustrum | | 611 | | 32 | 8 | -2 | 4.88 | | .01^a^ | |
|  | Temporoparietal junction (BA40) | | 467 | | 64 | -30 | 22 | 4.52 | | .002^b^ | |
|  | Posterior SCSR (BA25) | | 56 | | 2 | 14 | -6 | 3.59 | | .05^c^ | |
| *Replication:* | | | | | | | | | | | |
| Recurring episode MDD > stable remission MDD | | | | | | | | | | | |
|  | Ventrolateral putamen and claustrum | | 620 | | 32 | 8 | -2 | 4.78 | | .007^a^ | |
|  | Temporoparietal junction (BA40) | | 301 | | 64 | -30 | 22 | 4.17 | | .015^b^ | |
|  | Posterior SCSR (BA25) | | 76 | | 2 | 14 | -6 | 3.63 | | .04^c^ | |
| *No time and dispersion derivatives modelled:* | | | | | | | | | | | |
| Recurring episode MDD > stable remission MDD | | | | | |  |  |  | |  | |
|  | Ventrolateral putamen and claustrum | | | 620 | 32 | 8 | -2 | 4.79 | | .007^a^ | |
|  | Temporoparietal junction (BA40) | | | 302 | 64 | -30 | 22 | 4.17 | | .015^b^ | |
|  | Posterior SCSR (BA25) | | | 78 | 2 | 14 | -6 | 3.63 | | .04^c^ | |
| RSATL PPI effects for the recurring episode vs. the stable remission MDD group (self- vs other-blame emotions) as in the previous paper [Lythe et al. (2015)], and its replication, as well as a model with no time and dispersion derivatives were compared.^a^ Region surviving inclusive masking at uncorrected *p* = .001, with cluster-level FWE correction over the whole brain ^b^ Region surviving inclusive masking at uncorrected *p* = .005, with cluster-level FWE correction over the whole brain ^c^ Region surviving voxel-based FWE correction over the *a priori* SCSR using small-volume correction. BA = Brodmann Area; PPI = psychophysiological interaction; RSATL = right superior anterior temporal lobe; SCSR = subgenual cingulate cortex and adjacent septal region; MDD = major depressive disorder; FWE = family-wise error; MNI = Montreal Neurological Institute | | | | | | | | | | | |

# References

1. Green, S., et al., *Guilt-selective functional disconnection of anterior temporal and subgenual cortices in major depressive disorder.* Archives of General Psychiatry, 2012. **69**(10): p. 1014-21.

2. Grinband, J., et al., *Detection of time-varying signals in event-related fMRI designs.* Neuroimage, 2008. **43**(3): p. 509-20.

3. Handwerker, D.A., J.M. Ollinger, and M. D'Esposito, *Variation of BOLD hemodynamic responses across subjects and brain regions and their effects on statistical analyses.* Neuroimage, 2004. **21**(4): p. 1639-51.

4. Burle, B., et al., *Spatial and temporal resolutions of EEG: is it really black and white? A scalp current density view.* International Journal of Psychophysiology, 2015. **97**(3): p. 210-20.

5. Lau, E.F., C. Phillips, and D. Poeppel, *A cortical network for semantics: (de)constructing the N400.* Nature Reviews Neuroscience, 2008. **9**(12): p. 920-33.

6. Lythe, K.E., et al., *Subgenual activation and the finger of blame: individual differences and depression vulnerability.* Psychological Medicine, 2020: p. 1-9.

7. Brett, M., et al. *Region of interest analysis using an SPM toolbox*. in *8th International Conference on Functional Mapping of the Human Brain*. 2002. Sendai, Japan: Neuroimage.

8. Lythe, K.E., et al., *Self-blame-selective hyperconnectivity between anterior temporal and subgenual cortices and prediction of recurrent depressive episodes.* JAMA Psychiatry, 2015. **72**(11): p. 1119-26.

9. Eklund, A., T.E. Nichols, and H. Knutsson, *Cluster failure: why fMRI inferences for spatial extent have inflated false-positive rates.* PNAS, 2016. **113**(28): p. 7900-7905.

10. Flandin, G. and K.J. Friston, *Analysis of family-wise error rates in statistical parametric mapping using random field theory.* Human Brain Mapping, 2019. **40**(7): p. 2052-2054.

11. Murphy, K., J. Bodurka, and P.A. Bandettini, *How long to scan? The relationship between fMRI temporal signal to noise ratio and necessary scan duration.* NeuroImage, 2007. **34**(2): p. 565-74.

12. Rorden, C. and M. Brett, *Stereotaxic display of brain lesions.* Behavioural Neurology, 2000. **12**: p. 191-200.
